# Scheduling PID Attitude and Position Control Frequencies for Time-Optimal Quadrotor Waypoint Tracking under Unknown External Disturbances

**DOI:** 10.3390/s22010150

**Published:** 2021-12-27

**Authors:** Cheongwoong Kang, Bumjin Park, Jaesik Choi

**Affiliations:** Kim Jaechul Graduate School of AI, Korea Advanced Institute of Science and Technology (KAIST), Daejeon 34141, Korea; cw.kang@kaist.ac.kr (C.K.); bumjin@kaist.ac.kr (B.P.)

**Keywords:** waypoint tracking, external disturbance estimation, quadrotor control, reinforcement learning, deep learning, artificial intelligence

## Abstract

Recently, the use of quadrotors has increased in numerous applications, such as agriculture, rescue, transportation, inspection, and localization. Time-optimal quadrotor waypoint tracking is defined as controlling quadrotors to follow the given waypoints as quickly as possible. Although PID control is widely used for quadrotor control, it is not adaptable to environmental changes, such as various trajectories and dynamic external disturbances. In this work, we discover that adjusting PID control frequencies is necessary for adapting to environmental changes by showing that the optimal control frequencies can be different for different environments. Therefore, we suggest a method to schedule the PID position and attitude control frequencies for time-optimal quadrotor waypoint tracking. The method includes (1) a Control Frequency Agent (CFA) that finds the best control frequencies in various environments, (2) a Quadrotor Future Predictor (QFP) that predicts the next state of a quadrotor, and (3) combining the CFA and QFP for time-optimal quadrotor waypoint tracking under unknown external disturbances. The experimental results prove the effectiveness of the proposed method by showing that it reduces the travel time of a quadrotor for waypoint tracking.

## 1. Introduction

In recent years, the use of quadrotor has rapidly increased in various fields, including agriculture [1,2], rescue [3,4], delivery [5], inspection [6], and map construction [7]. A quadrotor, or drone, shows a versatile ability for various kinds of missions because of the simple structure, the simple use of control, and useful properties, such as VTOL (vertical take-off and landing).

Quadrotor waypoint tracking is defined as controlling quadrotors to follow the given waypoints. To complete time-critical missions, such as rescue and delivery, time-optimal quadrotor waypoint tracking is essential. Although PID control [8] is widely used for controlling robots, including quadrotors [9,10,11], it is hardly generalizable to various environments, such as various trajectories and dynamic external disturbances, which cannot be precisely modeled due to the uncertainties [12]. Existing PID control methods for quadrotors use a fixed position control frequency, where the position errors are fed to the PID periodically. However, in this work, we discover that different trajectories require different control frequencies to achieve time-optimal tracking control. We also show that different control frequencies are required as the external disturbances change.

There have been numerous efforts for adaptive control in environmental changes, including cascade control [13,14], finite-time control [15,16], coordinated control [17,18], PID gain scheduling [19,20], backstepping control [21,22], sliding mode control [23,24], external disturbance estimation [25,26], and compensation [27,28]. However, no existing studies consider finding the best control frequencies for time-optimal tracking control.

Therefore, we suggest a method for scheduling PID control frequencies for time-optimal quadrotor waypoint tracking in various environments under unknown external disturbances. First, we propose the Control Frequency Agent (CFA), a deep reinforcement-learning-based model to schedule the ratio of position and attitude control frequencies. Given the information at the current timestep, the CFA finds the best control frequencies for time-optimal waypoint tracking in various trajectories. Secondly, we suggest the Quadrotor Future Predictor (QFP), a neural-network-based model that predicts the next state of a quadrotor. Given the current state and action of a quadrotor, QFP predicts the next state of the quadrotor. Finally, we propose a way to compensate the external disturbances by combining CFA and QFP. Specifically, the current external disturbances are estimated by QFP, and CFA schedules control frequencies given the estimates as additional information. The proposed method is generalizable to various trajectories under various unknown external disturbances. To the best of our knowledge, none of the existing studies deal with scheduling PID control frequencies for improving quadrotor waypoint tracking control.

To evaluate the effectiveness of the proposed method, we make comparisons with a conventional PID control method, whose attitude and position control frequencies are fixed. For experiments, we use various trajectories with and without external disturbances. The methods are evaluated in terms of the total travel time taken to follow the given waypoints. The experimental results show that the proposed method outperforms the conventional PID controller, which uses a fixed control frequency, in waypoint tracking under external disturbances.

Our contributions are as follows: (1) discovering that the optimal control frequencies may vary in different environments, (2) a deep reinforcement-learning-based Control Frequency Agent (CFA) that adjusts the control frequencies of a quadrotor for time-optimal quadrotor waypoint tracking in various environments, (3) a neural-network-based Quadrotor Future Predictor (QFP) that estimates the next state of a quadrotor, and (4) the combination of CFA and QFP for time-optimal quadrotor waypoint tracking under various unknown external disturbances.

## 2. Related Work

PID control [8] is generally used in many industries because of its simplicity [9,11]. However, conventional PID control may not be applicable under dynamic external forces. There have been numerous studies for adaptive control in dynamic environmental changes, such as cascade control [13,14], finite-time control [15,16], coordinated control [17,18], PID gain scheduling [19,20], backstepping control [21,22], sliding mode control [23,24], external force estimation [25,26], and compensation [27,28]. However, none of the studies consider scheduling control frequencies. Since this paper is focused on finding the optimal control frequencies in different environments, the existing methods are not directly comparable. Instead, the proposed method can be combined with the existing methods for further improvements.

One of the prominent approaches for handling unknown external disturbances is estimating external disturbances [25,26] and compensating the estimated disturbance [21,27,28]. Similarly, we train the external force estimator to predict the next state of a quadrotor given the current state and action. Then, we compensate the estimated external disturbance by scheduling PID attitude and position control frequencies. Although we follow the model architecture that consists of an external force estimator and a compensator, none of the existing studies compensate the estimated force by adjusting PID control frequencies.

## 3. Background

### 3.1. Quadrotor Dynamics

**Figure 1:**
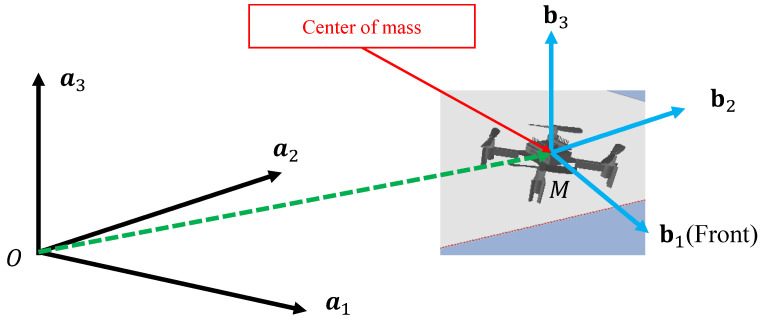
The description of the inertial frame A with triad $a_{1}$, $a_{2}$, and $a_{3}$ and the body frame B with triad $b_{1}$, $b_{2}$, and $b_{3}$. The position [x,y,z] is the vector from *O* to *M*.

The kinematics of a quadrotor can be described in an inertial frame A with a triad $a_{1}$, $a_{2}$, and $a_{3}$ and a body frame B with triad $b_{1}$, $b_{2}$, and $b_{3}$. The geometric view of two frames is presented in Figure 1. The quadrotor has 6 degrees of freedom in the position ξ=[x,y,z] and the orientation η=[ϕ,θ,ψ], where ξ is the position of the body frame with respect to the inertial frame A and η is the rotation coordinates of the body frame with respect to the inertial frame. These values are denoted as roll, pitch, and yaw [29].

We use the Z−X−Y Euler angle convention to model the rotation of a quadrotor in the inertial frame, following [29]. By Newton’s equations of motion, we have

(1) $$m\ddot{\xi }=\left[\begin{array}{c} 0\\ 0\\ -mg \end{array}\right]{+}^{A}R_{B}\left[\begin{array}{c} 0\\ 0\\ F_{1}+F_{2}+F_{3}+F_{4} \end{array}\right]$$

where *m* is the mass of the quadrotor, *g* is the gravity, and ${}^{A}R_{B}$ is the rotation matrix from A to B. Each $F_{i}$ is a vertical force produced by each rotor of the quadrotor with angular velocity $w_{i}$. A rotor also produces a moment $M_{i}$.

(2) $$\begin{array}{c} F_{i}=k_{F}w_{i}^{2} \end{array}$$

(3) $$\begin{array}{c} M_{i}=k_{M}w_{i}^{2} \end{array}$$

Here, $k_{F}$ and $k_{M}$ are constants that should be experimentally tuned. By summing the forces, we can control the acceleration of the quadrotor. Therefore, we have the first input $u_{1}$

(4) $$u_{1}=\sum_{i=1}^{4}F_{i}$$

Rotor 1 and 3 rotate in the $-b_{3}$ direction and produce moment $M_{1}$ and $M_{3}$, whose directions are opposite to the direction of rotation. Furthermore, rotor 2 and 4 rotate in the $b_{3}$ direction and produce moment $M_{2}$ and $M_{3}$. $M_{1}$ and $M_{3}$ act in the $b_{3}$ direction, while $M_{2}$ and $M_{4}$ act in the $-b_{3}$ direction. By Euler’s equations of motion, the angular acceleration is defined as

(5) $$I\left[\begin{array}{c} \dot{p}\\ \dot{q}\\ \dot{r} \end{array}\right]=\left[\begin{array}{c} L(F_{2}-F_{4})\\ L(F_{3}-F_{1})\\ M_{1}-M_{2}+M_{3}-M_{4} \end{array}\right]-\left[\begin{array}{c} p\\ q\\ r \end{array}\right]\times I\left[\begin{array}{c} p\\ q\\ r \end{array}\right]$$

where × is the outer product, *L* is the length of the blades, and *I* is the inertial matrix of the quadrotor. We have the second input vector $u_{2}$ using Equations (2) and () as follows.

(6) $$u_{2}=\left[\begin{array}{cccc} 0 & L & 0 & -L\\ -L & 0 & L & 0\\ k_{M}/k_{F} & -k_{M}/k_{F} & k_{M}/k_{F} & -k_{M}/k_{F} \end{array}\right]\left[\begin{array}{c} F_{1}\\ F_{2}\\ F_{3}\\ F_{4} \end{array}\right]$$

The control of a quadrotor can be separated into the position and the attitude control. In general, attitude control is ≈10 times more frequent than the attitude control [30]. The implementation of the stable control keeps the attitude of the drone as [0,0,ψ] while controlling the position of the drone to follow the reference trajectory. The hover configuration is given as $\xi =\xi_{0}$, θ=ϕ=0, $\dot{\xi }=0$ and $\dot{\phi }=\dot{\psi }=\dot{\theta }=0$. In this state, the force for each robot is $\frac{mg}{4}$.

### 3.2. PID Control

**Figure 2:**
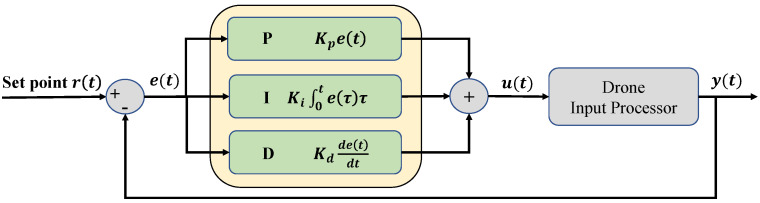
The basic PID controller. The error term $e_{t}$ is the difference between the desired position r(t) and the sensed position y(t). Each PID term is computed based on the current and past errors. The force of each rotor is computed from Equations (4) and (6). After processing the input, we have the current output y(t). This feedback loop decreases $e_{t}$ gradually.

When there is an error e(t) at a time step *t* between the sensed position y(t) and the desired position r(t), the controller calculates the motor speed to reduce the error to 0. When the control only uses the current error, it usually fails to reach the desired setpoint, and thus, integral and derivative terms are additionally required. A Proportional-Integral-Derivative (PID) controller has three gains. The proportional term is used to reduce the currently observed error. The integral term is used to accumulate errors to reach the desired position faster. The derivative term is used to stabilize the control. The overall PID control process is shown in Figure 2.

### 3.3. Position and Attitude Control

**Figure 3:**
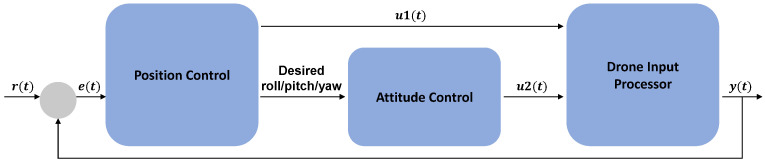
The PID feedback loop for position and attitude control of a quadrotor. Given the desired point r(t) and sensed point y(t), the error term e(t) is computed and fed to the position controller. Next, the position controller produces a control input u1(t) and determines the desired roll, pitch, and yaw angles. Then, the attitude controller produces a control input u2(t) given the desired angles. The control inputs u1(t) and u2(t) are fed to the drone input processor to control the rotors.

For the quadrotor control, the position and attitude control can be separated. Therefore, it is possible to run the attitude control more frequently than the position control using the nested feedback loops. An example of the feedback loop is illustrated in Figure 3.

The position control is to follow the reference trajectory in three dimensions. Additionally, the desired yaw angle can be specified independently. For position control, PID coefficients must be properly tuned for each dimension and yaw angle. Then, the position control algorithm will produce the desired roll and pitch angles.

The attitude control is to track a trajectory following the desired roll, pitch, and yaw angles. The attitude should approach the nominal hover state, where the roll and pitch are near zero. PID coefficients must be properly determined for each of the raw, pitch, and yaw angles.

## 4. Method

**Figure 4:**
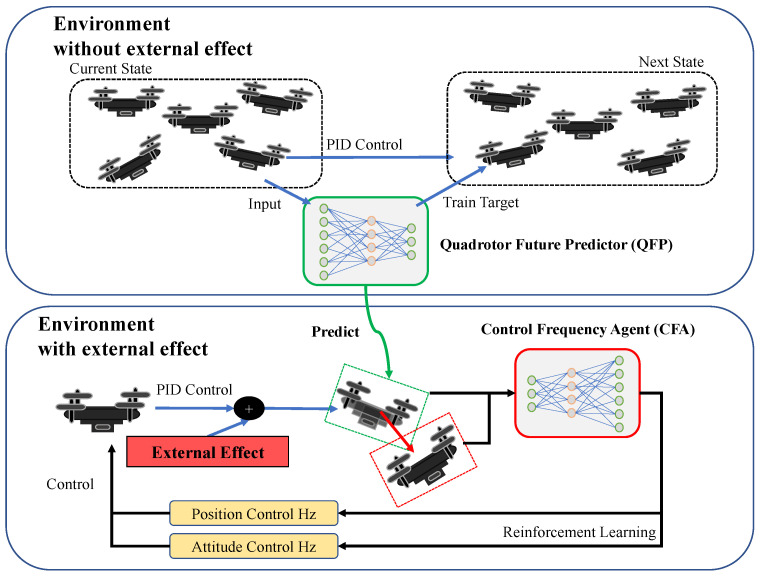
The proposed QFP and CFA models. QFP is trained to predict the next position of a quadrotor controlled by PID in the environment without external effects. After training, the next state estimated by QFP is used as the input of CFA. Then, CFA is trained to adjust the ratio of the position and attitude control frequencies against external effects by maximizing the given reward function. The approximated external effect is computed as the difference between the current state (in a red dashed box) and the estimated state (in a green dashed box). CFA is trained to balance the position and attitude control frequencies for time-optimal waypoint tracking control.

In this section, we propose a method to schedule PID control frequencies for time-optimal quadrotor waypoint tracking under unknown external disturbances. The proposed method includes (1) the Control Frequency Agent (CFA), (2) the Quadrotor Future Predictor (QFP), and (3) combining CFA and QFP (Figure 4).

### 4.1. Control Frequency Agent

A quadrotor mainly has two controllers, an attitude controller and a position controller. The inner attitude control loop controls the Euler angles and the outer position control loop controls the trajectory in three dimensions. In general, the attitude control frequency is 5∼10 times larger than the position control frequency [29]. As the attitude control frequency becomes larger than the position control frequency, the quadrotor flies more stably, but it follows the target position slowly. On the other hand, as the position control frequency becomes larger than the attitude control frequency, the flight becomes unstable, so it may fall. In addition, we find that the optimal control frequencies may vary according to the environmental changes. Therefore, finding the optimal ratio of control frequencies is essential for time-optimal control in quadrotor waypoint tracking.

Therefore, we propose a Control Frequency Agent (CFA), which is a deep reinforcement-learning-based model that schedules the ratio of control frequencies of the position and attitude of the quadrotor. We define the observation as the current state of a quadrotor, which includes rotations in roll, pitch, yaw angles, a linear and angular velocity vector, and the motors’ speeds. The action space is defined as a discrete action space with three possible actions: (1) increase the attitude frequency, (2) decrease the attitude frequency, and (3) keep the current frequency ratio. The reward is the sparse reward and the agent obtains 10/(1+reachtime) when it reaches the last waypoint. We use a conventional implementation of the on-policy model for the CFA model. Although we adopt the Proximal Policy Gradient [31] for its simplicity, any deep reinforcement learning models can be adopted. The model is trained to maximize the reward, which is maximized when the total travel time taken to follow the reference waypoints is minimized.

### 4.2. Quadrotor Future Predictor

Although PID control is simple and effective, the input of the controller is derived from the linear equation of quadrotor’s kinematic information, not considering external forces. Therefore, we suggest a way to separate the total setpoint error in the error caused by the PID controller and the error caused by the external effects. Let $SP_{t}$ and $CP_{t}$ be the set point and the current position of the quadrotor at a timestep *t*. The PID controller computes the target RPMs for each rotor, and the quadrotor moves to the next position $CP_{t+1}$. Here, the error is defined as $E_{t}=SP_{t}-CP_{t+1}$ if and only if there is no external effect. However, when there are external effects, the current position $CP_{t+1}$ becomes $CP_{t+1}^{w}=CP_{t+1}+\epsilon_{t}$, where $\epsilon_{t}$ is the additional movement of the drone by external effects. The error $E_{t}^{w}$ in the environment with the external effects can be written as

(7) $$\begin{array}{cc} E_{t}^{w} & =SP_{t}-CP_{t+1}^{w} \end{array}$$

(8) $$\begin{array}{cc} \; & =SP_{t}-\left(CP_{t+1}+\epsilon_{t}\right) \end{array}$$

(9) $$\begin{array}{cc} \; & =\left(SP_{t}-CP_{t+1}\right)+\epsilon_{t} \end{array}$$

(10) $$\begin{array}{c} =E_{t}+\epsilon_{t} \end{array}$$

Therefore, the error term can be decomposed into the error term $E_{t}$ produced by the PID control and the error $\epsilon_{t}$ produced by the external effects. Even though PID control is simple, predicting the next position of the quadrotor controlled by the PID control is not trivial. This is because PID control is dependent not only on the current position but also on the past information of the control.

Based on the setpoint error decomposition defined above, we propose the Quadrotor Future Predictor (QFP), which predicts the next state of the drone based on (1) the kinematic information of the quadrotor, including $CP_{t}$ and $SP_{t}$, (2) the current PID errors before multiplying gains, and (3) the gain for each PID term. The model is trained to predict the next state of the drone, which includes the next position $CP_{t+1}$ and the Euler angles. Specifically, the training samples are generated by moving to a random position near the current position, where the random position is within the range of where the quadrotor can move in a single timestep. The model is composed of three feedforward layers with a ReLU activation function in-between. Batch normalization and dropout are applied after the first and second layers. The observation of the QFP is defined as the current state of a quadrotor, including roll, pitch, yaw angles, the linear and angular velocity vector, and the motors’ speeds. The QFP estimates a relative vector of the next position from the current position. Specifically, the QFP model is trained with mean squared error (MSE) loss to predict the next position ${\hat{CP}}_{t+1}$ of the quadrotor based on the current state information.

(11) $$\begin{array}{cc} CP_{t+1}^{w} & =CP_{t+1}+\epsilon_{t} \end{array}$$

(12) $$\begin{array}{c} \;\approx {\hat{CP}}_{t+1}+\epsilon_{t} \end{array}$$

Since $CP_{t+1}^{w}$ is an observed value, we can approximate $\epsilon_{t}\approx CP_{t+1}^{w}-{\hat{CP}}_{t+1}$. Accordingly, we can approximate the external effect with the QFP model. Note that the model is trained in the environment where there is no external effect.

### 4.3. Trajectory Tracking with CFA + QFP

**Figure 5:**
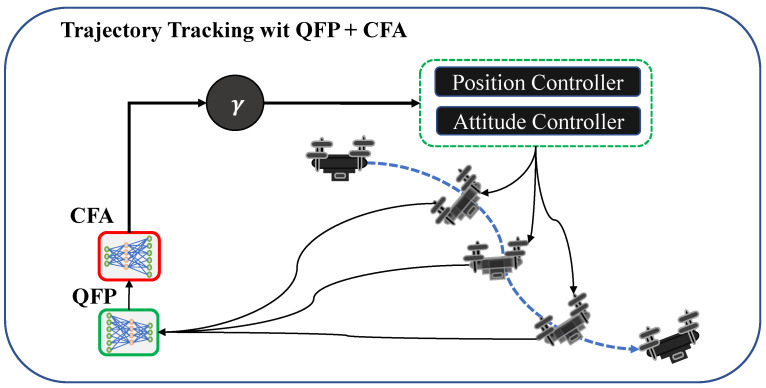
An illustration of trajectory tracking with the QFP+CFA model. At each time step, the QFP model predicts the next state of the quadrotor controlled by the PID, and the CFA model outputs a ratio γ to balance the position and attitude control frequencies.

After training both the QFT and CFA models, it is possible to use a feedback loop that combines the QFP, CFA, and a conventional PID controller for waypoint tracking. The overall procedure is illustrated in Figure 5. Given the current state of a drone and the motor RPMs computed by the PID controller, the QFP model predicts the next state and approximates the external disturbances. Then, given the estimated external effects as additional information, the CFA model adjusts the position and attitude control frequencies periodically. Then, the PID controller runs with the adjusted control frequencies.

## 5. Experiments

We use the the *gym-pybullet-drones* [32] drone simulation platform to conduct experiments. It is an open-source, OpenAI Gym-like [33] multi-quadrotor simulator based on the Pybullet Physics [34]. We use the Bitcraze Crazyfile 2.x, which is one of the default quadrotor models.

The QFP model comprises three layers of a fully connected neural network with ReLU activation. The sizes of the hidden dimensions are 16, 32, and 64, respectively, for each layer. To train the QFP model, we use the 4803 samples for training and 24,019 samples for validation that are generated by random moves. For hyper-parameters, we use the Adam optimizer with the learning rate of 0.001, the dropout probability of 0.2, the batch size of 256, and the number of epochs as 100. The CFA model is composed of three fully connected layers with ReLU activation. The sizes of the hidden dimensions are tested with 256, 256, and 256, respectively, for each layer. For training the CFA model, we use the batch size of 256, the horizon size of 4000, the lambda as 0.99, the gamma as 0.99, the learning rate of 0.00001, and the number of epochs as 1000. Each episode is 10 seconds long at maximum. We compare the proposed model (CFA+QFA) with the conventional PID baseline and the CFA without QFA model (CFA-QFA). The CFA-QFA adjusts the position control frequency without information about the external effects estimated by the QFA model. By comparing the CFA+QFA model with the CFA-QFA model, we verify the effectiveness of the QFA model. Finally, we show the effectiveness of combining the QFP and CFA models by comparing the conventional PID and the CFA+QFA model.

**Figure 6:**
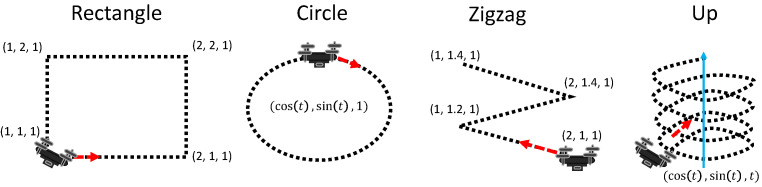
Four types of trajectories for the test of position frequency.

We first empirically show that the optimal PID position frequency may vary for different trajectory types. We show the results on the four trajectory types shown in Figure 6. The first three of them are 2D movements, and the last one is 3D. The distances between waypoints are equally distributed. When the quadrotor is close enough to the current target waypoint, the target waypoint is set to the next waypoint. Next, we measure the prediction performance of the QFP model. We then compare the performance of the CFA+QFP, CFA-QFP, and PID models in waypoint tracking in various environments.

## 6. Results

**Figure 7:**
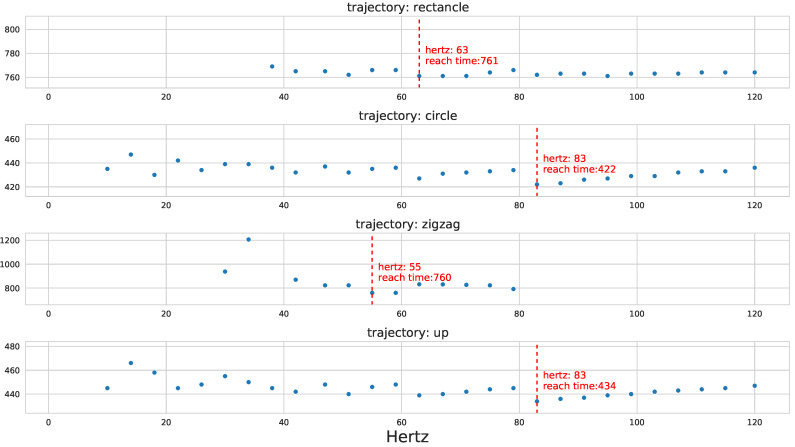
The results of best position frequencies for the four trajectories. X-axis represents a frequency (in hertz) for the position control, and Y-axis represents the reach time of the last waypoint. The dashed red line indicates the optimal position frequency that minimizes the reach time.

First, we test the effect of the position control frequencies for the four different trajectory types. The results are shown in Figure 7. We discover that the optimal control frequencies vary for different trajectories. Specifically, the rectangle and zigzag types require smaller position control frequencies than the circle and up types to minimize the reach time. In addition, there are some cases where the position control frequency significantly affects the reach time, as shown in the third row (zigzag).

**Figure 8:**
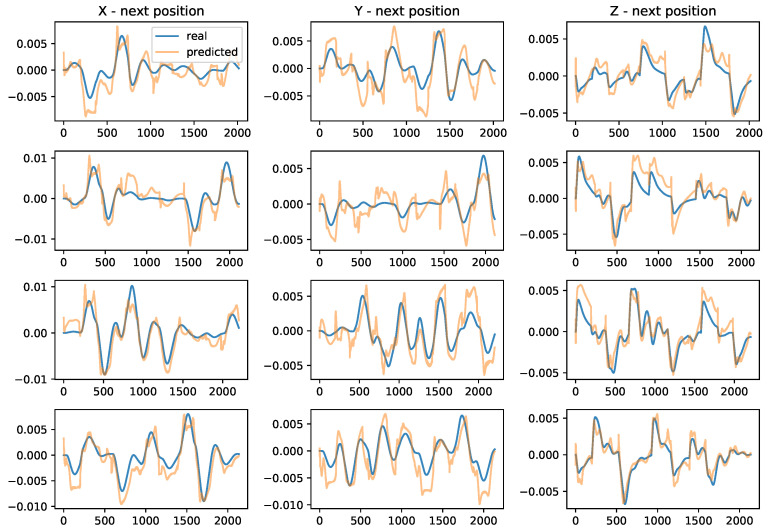
The performance of QFP for the four random trajectories. Each row represents one of the four trajectories, respectively. X-axis is a time step, and Y-axis is a relative position (next position minus current position). These results show that the QFP model predicts the direction of the next position perfectly, whereas there is a little gap in magnitude.

Next, we show the results of the QFP performance for four randomly generated trajectories in Figure 8. The blue line indicates the real trajectory, and the orange line indicates the predicted trajectory. We observe that the QFP model perfectly predicts the direction of the next position, whereas there are some errors in the magnitude. However, it is sufficient statistics because it is important for a quadrotor to know the direction of external forces, such as drag and wind.

**Figure 9:**
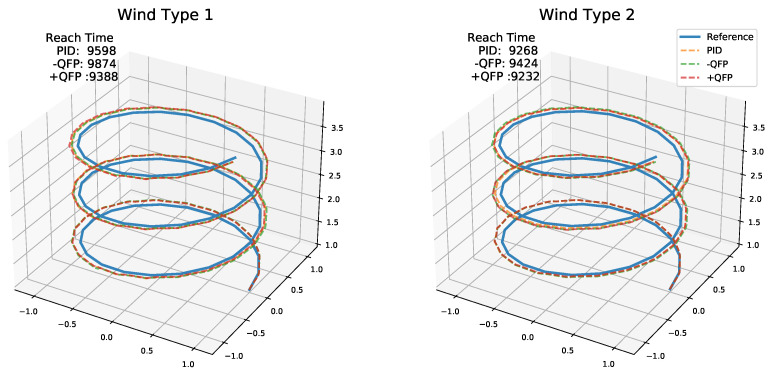
The result trajectory of the quadrotor for models PID, CFA-QFP, and CFA+QFP. Wind type 1 is the circular wind, and wind type 2 is the line wind. The number is the reach time of the last waypoint.

**Figure 10:**
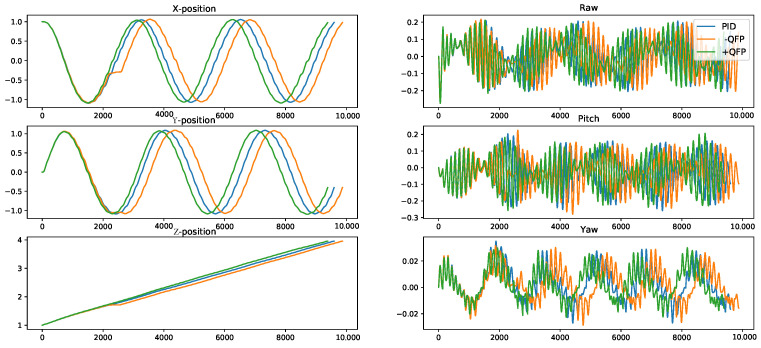
The position and the attitude angles of the quadrotor of the up-trajectory with circular wind type. The X-axis is the time step. The CFA+QFP model reached the last waypoint first.

**Figure 11:**
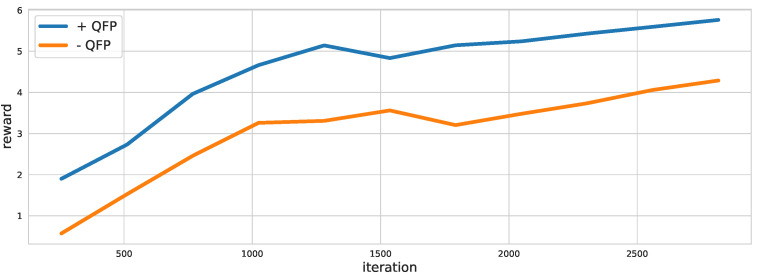
The reward graph of the CFA+QFP and CFA-QFP models. The reward graph shows that the disturbance information predicted from the QFA is useful to the CFA model.

Then, to verify the effectiveness of the proposed method (CFA+QFP) under unknown external disturbances, we test the performance of the CFA+QFP model under different wind types. Specifically, the results on the up-trajectory with two different types of wind are shown in Figure 9 and Figure 10. We compare the proposed method (CFA+QFP) with the conventional PID controller and CFA-QFP, which uses the CFA model without the external effects estimated by the QFP model. Figure 9 shows the results of the trajectory and the reach time of the last waypoint. The first wind type is a circular wind type, which is not trivial to estimate the external effects. The second wind type is a linear wind type, which is simpler than the first one. The results show that the proposed method outperforms the conventional PID controller by scheduling the PID control frequencies. We also observe that the CFA performs worse than the conventional PID controller without the QFP model. This stipulates that the external effects estimated by the QFP model are essential for finding the optimal control frequency against the external disturbances. Additionally, we observe that the performance gap is larger in the first wind type, which is more complex. This indicates that the proposed model is more effective as the environment changes become more complex. For more detailed analysis, Figure 10 shows the XYZ-positions and raw, pitch, and yaw angles on the first wind type. We see that the CFA-QFP model fails to find the optimal control frequency without the information estimated by the QFP model, and so, it fails to stably follow the waypoints at some point. We also show the average reward graph for two models for episodes in Figure 11. Although the reward of the CFA-QFP increases, there is a gap between the reward of CFA+QFP and that of CFA-QFP. This is because the estimated external effects are essential information to find the optimal control frequency.

## 7. Conclusions

In this work, we discover that the optimal control frequencies may vary in different environments. Accordingly, we suggest a method for scheduling PID control frequencies for time-optimal quadrotor waypoint tracking in various environments under unknown external disturbances. The proposed method is composed of (1) the Control Frequency Agent (CFA), which adjusts the PID control frequencies according to environmental changes based on deep reinforcement learning, and (2) the Quadrotor Future Predictor (QFP), which estimates the external disturbances based on neural networks. Combining the CFA and QFP, the proposed method finds the best PID control frequencies for time-optimal quadrotor waypoint tracking under various environmental changes. The experimental results verify that the proposed method outperforms the conventional PID controller in waypoint tracking in various environments. 

## Data Availability

You can find the code implementation in https://github.com/fxnnxc/mdpi_drone_control (accessed on 27 December 2021).

## References

[1] Radoglou-Grammatikis P., Sarigiannidis P., Lagkas T., Moscholios I. (2020). A compilation of UAV applications for precision agriculture. Comput. Netw..

[2] Tokekar P., Vander Hook J., Mulla D., Isler V. (2016). Sensor planning for a symbiotic UAV and UGV system for precision agriculture. IEEE Trans. Robot..

[3] Schedl D.C., Kurmi I., Bimber O. (2021). An autonomous drone for search and rescue in forests using airborne optical sectioning. Sci. Robot..

[4] Lygouras E., Santavas N., Taitzoglou A., Tarchanidis K., Mitropoulos A., Gasteratos A. (2019). Unsupervised human detection with an embedded vision system on a fully autonomous UAV for search and rescue operations. Sensors.

[5] Lee J. Optimization of a modular drone delivery system. Proceedings of the 2017 Annual IEEE International Systems Conference (SysCon).

[6] Besada J.A., Bergesio L., Campaña I., Vaquero-Melchor D., López-Araquistain J., Bernardos A.M., Casar J.R. (2018). Drone mission definition and implementation for automated infrastructure inspection using airborne sensors. Sensors.

[7] Schmuck P., Chli M. Multi-UAV collaborative monocular SLAM. Proceedings of the 2017 IEEE International Conference on Robotics and Automation (ICRA).

[8] Ang K.H., Chong G., Li Y. (2005). PID control system analysis, design, and technology. IEEE Trans. Control Syst. Technol..

[9] Bouabdallah S., Siegwart R. Full control of a quadrotor. Proceedings of the 2007 IEEE/RSJ International Conference on Intelligent Robots and Systems.

[10] Salih A.L., Moghavvemi M., Mohamed H.A., Gaeid K.S. (2010). Flight PID controller design for a UAV quadrotor. Sci. Res. Essays.

[11] Li J., Li Y. Dynamic analysis and PID control for a quadrotor. Proceedings of the 2011 IEEE International Conference on Mechatronics and Automation.

[12] Liu H., Zhao W., Zuo Z., Zhong Y. (2016). Robust control for quadrotors with multiple time-varying uncertainties and delays. IEEE Trans. Ind. Electron..

[13] Szafranski G., Czyba R. (2011). Different approaches of PID control UAV type quadrotor. Proceedings of the International Micro Air Vehicle Conference and Competitions 2011 (IMAV 2011).

[14] Wang N., Su S.F., Han M., Chen W.H. (2018). Backpropagating constraints-based trajectory tracking control of a quadrotor with constrained actuator dynamics and complex unknowns. IEEE Trans. Syst. Man Cybern. Syst..

[15] Zhu W., Du H., Cheng Y., Chu Z. (2017). Hovering control for quadrotor aircraft based on finite-time control algorithm. Nonlinear Dyn..

[16] Wang N., Deng Q., Xie G., Pan X. (2019). Hybrid finite-time trajectory tracking control of a quadrotor. ISA Trans..

[17] Do K.D. (2015). Coordination control of quadrotor VTOL aircraft in three-dimensional space. Int. J. Control.

[18] Wang N., Ahn C.K. (2021). Coordinated Trajectory Tracking Control of a Marine Aerial-Surface Heterogeneous System. IEEE/ASME Trans. Mech..

[19] Åström K.J., Hägglund T., Hang C.C., Ho W.K. (1993). Automatic tuning and adaptation for PID controllers—A survey. Control Eng. Pract..

[20] Thanh V.N., Vinh D.P., Nam L.H., Nghi N.T., Le Anh D. Reinforcement Q-learning PID Controller for a Restaurant Mobile Robot with Double Line-Sensors. Proceedings of the 4th International Conference on Machine Learning and Soft Computing.

[21] Chen F., Jiang R., Zhang K., Jiang B., Tao G. (2016). Robust backstepping sliding-mode control and observer-based fault estimation for a quadrotor UAV. IEEE Trans. Ind. Electron..

[22] Zhao W., Meng Z., Wang K., Zhang H. (2021). Backstepping Control of an Unmanned Helicopter Subjected to External Disturbance and Model Uncertainty. Appl. Sci..

[23] Mofid O., Mobayen S., Wong W.K. (2020). Adaptive terminal sliding mode control for attitude and position tracking control of quadrotor UAVs in the existence of external disturbance. IEEE Access.

[24] Thanh H.L.N.N., Lee C.H., Hong S.K. Adaptive Perturbation Estimator based Dynamic Control using PID Sliding Manifold for a Quadcopter UAV. Proceedings of the 2021 International Conference on Advances in Electrical Computing, Communication and Sustainable Technologies (ICAECT).

[25] Chen F., Lei W., Zhang K., Tao G., Jiang B. (2016). A novel nonlinear resilient control for a quadrotor UAV via backstepping control and nonlinear disturbance observer. Nonlinear Dyn..

[26] Tomić T., Ott C., Haddadin S. (2017). External wrench estimation, collision detection, and reflex reaction for flying robots. IEEE Trans. Robot..

[27] Wang Y., Sun J., He H., Sun C. (2019). Deterministic policy gradient with integral compensator for robust quadrotor control. IEEE Trans. Syst. Man Cybern. Syst..

[28] Pi C.H., Ye W.Y., Cheng S. (2021). Robust quadrotor control through reinforcement learning with disturbance compensation. Appl. Sci..

[29] Valavanis K.P., Vachtsevanos G.J. (2015). Handbook of Unmanned Aerial Vehicles.

[30] Gurdan D., Stumpf J., Achtelik M., Doth K.M., Hirzinger G., Rus D. Energy-efficient autonomous four-rotor flying robot controlled at 1 kHz. Proceedings of the 2007 IEEE International Conference on Robotics and Automation.

[31] Schulman J., Wolski F., Dhariwal P., Radford A., Klimov O. (2017). Proximal policy optimization algorithms. arXiv.

[32] Panerati J., Zheng H., Zhou S., Xu J., Prorok A., Schoellig A.P. (2021). Learning to Fly—A Gym Environment with PyBullet Physics for Reinforcement Learning of Multi-agent Quadcopter Control. arXiv.

[33] Brockman G., Cheung V., Pettersson L., Schneider J., Schulman J., Tang J., Zaremba W. (2016). Openai gym. arXiv.

[34] Coumans E., Bai Y. (2016). Pybullet, a Python Module for Physics Simulation for Games, Robotics and Machine Learning. https://docs.google.com/document/d/10sXEhzFRSnvFcl3XxNGhnD4N2SedqwdAvK3dsihxVUA/edit#heading=h.2ye70wns7io3.

